# Fraction-Seq: An Integrated Experimental and Computational Workflow for Determining the Localization and Abundance of Small Non-Coding RNAs in Subcellular Compartments

**DOI:** 10.3390/ncrna12040029

**Published:** 2026-08-05

**Authors:** Siddhartha Shah, Tess Cherlin, Yi Jing, Stepan Nersisyan, Benjamin Leiby, Isidore Rigoutsos

**Affiliations:** 1 Computational Medicine Center, Thomas Jefferson University, Philadelphia, PA 19017, USA; 2 Division of Biostatistics and Bioinformatics, Thomas Jefferson University, Philadelphia, PA 19017, USA

**Keywords:** small non-coding RNAs (sncRNAs), microRNAs (miRNAs), miRNA isoforms (isomiRs), tRNA-derived fragments (tRFs), rRNA-derived fragments (rRFs), Y RNA-derived fragments (yRFs), Fraction-seq

## Abstract

Small non-coding RNAs (sncRNAs) have garnered considerable attention in recent years, following accumulating evidence of their critical roles in many cellular processes. Among sncRNAs, microRNAs (miRNAs) and their isoforms (isomiRs), tRNA-derived fragments (tRFs), rRNA-derived fragments (rRFs), and Y RNA-derived fragments (yRFs) account for more than 95% of all sncRNAs found in cells. Despite their critical regulatory roles, most sncRNAs remain uncharacterized because their functionalization is a lengthy and challenging undertaking. Knowing an sncRNA’s abundance helps prioritize among the various choices, while knowing where it localizes in the cell greatly limits the number and identity of its potential targets and helps understand its function. Most studies to date have assumed that the subcellular localization of the various sncRNA classes is understood and remains unchanged across cell types. However, as we have recently demonstrated, the subcellular distribution of sncRNAs follows complex patterns that depend on the sequence of the sncRNA, the presence or absence of post-transcriptionally added non-templated nucleotides, and the cell type. To determine the subcellular localization and abundance of sncRNAs and aid the design of targeted experimental studies of sncRNA function, we developed “Fraction-seq.” The method combines an experimental and an analytical component to remove cross-fraction contamination and reconstruct the true abundance of sncRNAs in each considered fraction. Fraction-seq can be applied to any adherent cells from any organism without modifications and can reconstruct the abundance of all categories of sncRNAs. Accompanying this detailed protocol is a newly developed port of the original SAS codes to the widely used R programming language. The codes and an example are freely available through our center’s GitHub page.

## 1. Introduction

Deep sequencing has enabled the discovery of many categories of small non-coding RNAs (sncRNAs), including microRNAs (miRNAs) and their isoforms (isomiRs) [1,2,3], tRNA-derived fragments (tRFs) [4,5], rRNA-derived fragments (rRFs) [6,7], and Y RNA-derived fragments (yRFs) [8,9] in many contexts. Together, these four categories account for more than 95% of all sncRNAs found in cells [8,10]. We note here that the presentation emphasizes isomiRs, tRFs, rRFs, and yRFs because these classes have been extensively studied by many groups for many years and have intriguing properties that we discuss below. However, the Fraction-seq method we present can be applied without changes to any type of sncRNA present in the sequencing data the user wishes to analyze. The method only requires that the various sncRNAs be given unique identifiers, a topic that we also discuss in detail below.

For many years, isomiRs, tRFs, rRFs, and yRFs were either considered aberrant transcripts and ignored or, in the case of isomiRs, viewed as correlated and their abundances combined before any downstream analyses. It is now understood that sncRNAs belonging to these classes have critical regulatory roles [11,12,13,14,15,16], are involved in transgenerational inheritance [17,18], and regulate protein translation [19,20]. Notably, their abundance depends on personal attributes (e.g., sex, ancestry, age) [6,21,22,23,24], tissue type [1,4,7,25], and disease [1,8,25,26]. Co-expressed sncRNAs arising from the same parental RNA (“siblings”) are generally uncorrelated [11,25,27], and can target different genes [11,12,28] in patients who differ by ancestry [23]. These dependencies suggest a complex molecular milieu underlying diseases and disorders, underscoring the direct relevance of sncRNAs for studying disparities and realizing precision medicine [23,24,25].

The genomes in the nucleus and the mitochondrion (MT) encode transcripts whose processing produces isomiRs, tRFs, rRFs, and yRFs, which collectively account for more than 95% of all sncRNAs found in cells [8,9,10]. Nucleus-encoded transcripts produce isomiRs, tRFs, rRFs, and yRFs, whereas MT-encoded transcripts produce tRFs and rRFs. As the sncRNA field evolved, the assumption that the subcellular localization of these sncRNAs is well understood and remains unchanged across cell types persisted. Namely, miRNAs/isomiRs and tRFs from nucleus-encoded tRNAs were believed to localize to the cytoplasm [5,29,30], as were rRFs from nucleus-encoded rRNAs [15]. Analogously, tRFs and rRFs derived from MT-encoded tRNAs and rRNAs, respectively, were believed to remain in the MT. However, a few early studies began reporting exceptions. For example, several groups showed miRNAs and isomiRs that function in the nucleus [31,32,33,34], miRNAs that function in the MT [35,36], nucleus-encoded tRNAs and rRNAs that are imported to the MT [37,38], and MT-encoded tRNAs that are exported to the cytoplasm [39]. More recently, a large-scale study of tRFs across more than 10,000 human samples provided extensive evidence of nucleus-encoded and MT-encoded tRFs localizing to the nucleus, the cytoplasm, or the MT in various combinations [25].

**Figure 1:**
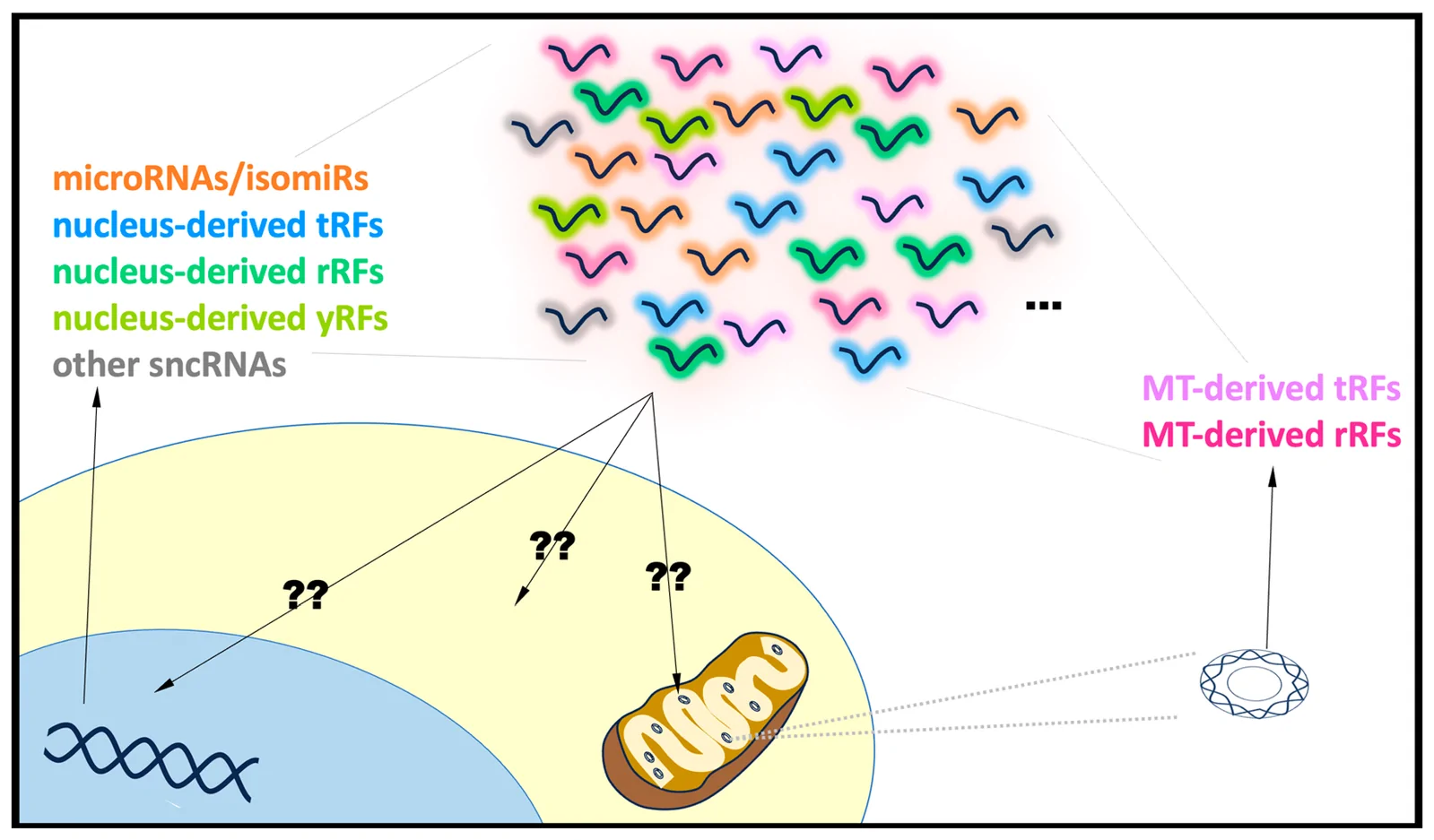
Pictorial summary of the problem at hand. Where in the cell do the sncRNAs produced from the processing of nucleus-encoded and MT-encoded transcripts go?

Figure 1 captures the problem at hand. Are the prevailing assumptions accurate, and the reported exceptions just that, i.e., exceptions? Or is the set of rules governing the subcellular localization of sncRNAs more complex? Additionally, if the latter, are there any dependencies? These questions prompted us to develop Fraction-seq, a method that facilitates the study of the subcellular localization of sncRNAs. To develop the method’s prototype, we considered the dependencies of sncRNA expression mentioned above and focused on three cell lines (BT-20, MDA-MB-231, MDA-MB-468) from the same tissue (breast) and same-sex donors (women) that modeled the same disease type (triple-negative breast cancer). These three cell lines have been used in experiments described in more than 10,000 peer-reviewed articles, underscoring the importance of our novel findings regarding the subcellular localization of sncRNAs [3] and the need for a method like Fraction-seq.

Cross-fraction contamination makes it difficult to obtain pure subcellular fractions, complicating sncRNA subcellular localization studies. The use of protein-based assays or northern blots to confirm the absence of contamination is limited by their sensitivity. Analogously, RNA-based assays also pose challenges, given the now-documented intracellular trafficking of sncRNAs, tRNAs, rRNAs, and their fragments. We note that “reference” non-coding RNAs, such as the *nuclear* marker U1, can pose challenges as well. Indeed, U1’s presence in the *cytoplasm* was first reported forty years ago [40], an observation that has since been confirmed by traditional methods [41,42] as well as by ENCODE’s deep-sequencing-based profiling of multiple cell lines [43].

Our proposed solution, Fraction-seq, couples a fractionation method with deep sequencing and an analytical method [3]. The latter leverages deep-sequencing data from biological replicates of multiple cellular fractions to estimate cross-fraction contamination and technical errors, then removes both to accurately recover the abundance of each sncRNA within each subcellular fraction.

Fraction-seq generated evidence that sncRNAs exhibit complex subcellular distribution patterns [3], which depend on the actual nucleotide sequence, the cell type, and whether the sncRNA is non-templated, i.e., has been modified through the post-transcriptional addition of nucleotides. We experimentally corroborated several of Fraction-seq’s findings, and also found that the complex subcellular trafficking patterns extend to sncRNAs that are longer than the 18–40 nucleotides (nts) typical of isomiRs, tRFs, rRFs, and yRFs. In the following, we present detailed instructions for carrying out the experimental and analytical portions of the Fraction-seq protocol.

## 2. Fraction-Seq: Method Overview

**Figure 2:**
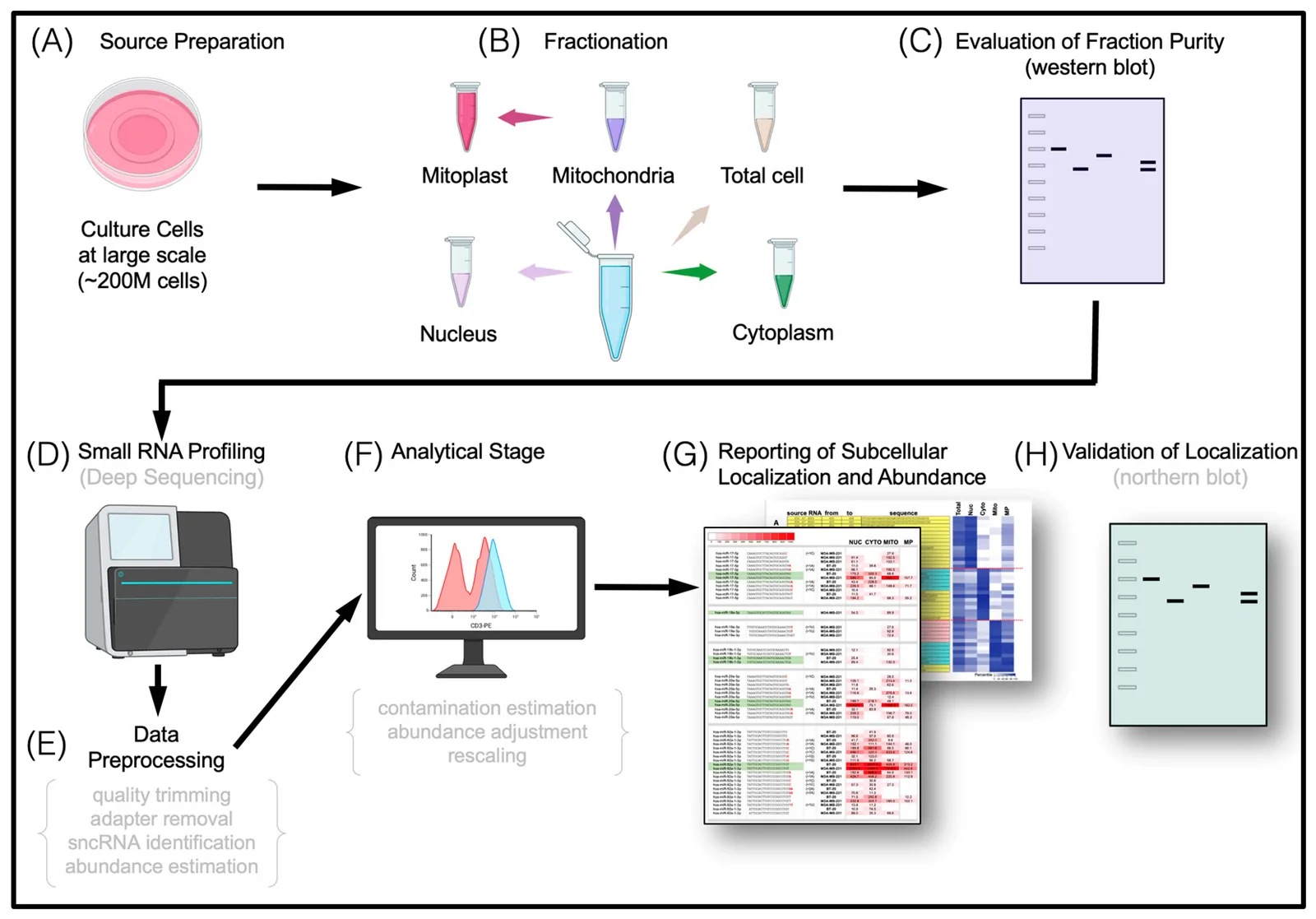
Outline of the Fraction-seq method. The images of step (G) were adapted from our earlier publication [3]. The remaining portions of this image were generated using BioRender.com.

Fraction-seq combines a novel experimental component with a novel analytical component, with the two components complementing one another. First, we present the experimental component (fractionation), followed by the analytical component (mixed-effects modeling). We recommend a minimum of three biological replicates, ideally five, for each cell line of interest. The number of replicates applies to total RNA and each of the nuclear, cytoplasmic, MT, and mitoplast fractions. Figure 2 summarizes the steps of the method.

Fraction-seq has undergone one key improvement since its recent publication [3]: we ported the original SAS codes to the widely used R programming language and made these updated codes available through GitHub. On a related note, we clarify that the analytical component of the method applies to *all types* of sncRNAs that may be present in a biological sample, i.e., it is not exclusive to isomiRs, tRFs, or rRFs. While miRNAs/isomiRs, tRFs, and rRFs have commanded the lion’s share of attention to date, other publications have also shown the relevance of these analyses for yRFs [8,9,10,18,44] and PIWI-interacting RNAs (piRNAs) [45,46,47], among other types, in humans and other organisms [48]. The category to which an sncRNA belongs need not be known; assigning a unique identifier to it is the only requirement.

### 2.1. Materials

Use cultured human cells as the starting material with roughly 200 million cells per biological replicate. To preserve their membrane integrity and the integrity of the subcellular fractions, cells need to be harvested at high density but should not be overconfluent. All subcellular fractions should be derived from the same initial cell population.

### 2.2. Reagents, Consumables, and Tools

Use of commercially available reagents for the isolation of cytoplasmic, MT, mitoplast, and nuclear fractions is recommended. The MT fractions are isolated using the Qiagen Mitochondrial Isolation Kit (Cat.#37612, Qiagen, Hilden, Germany). The nuclear fractions are isolated using the Thermo Fisher NE-PER™ Nuclear and Cytoplasmic Extraction Reagents kit (Cat.#78835, Thermo Fisher Scientific, Rockford, IL, USA). RNA extraction is conducted using Invitrogen’s TRIzol reagent (Cat.#15596018, Invitrogen, Carlsbad, CA, USA), and protein-based validation is conducted using the ProteinSimple JESS system (Cat.#004-650, Biotechne, Minneapolis, MN, USA). Sequencing library preparation uses New England Biolabs’ NEBNext^®^ Small RNA Library Prep Set for Illumina (Cat.#E7330, New England Biolabs, Ipswich, MA, USA). Detection of sncRNAs by northern blotting and validation of sequencing-based findings uses the Roche DIG Oligonucleotide 3′-End Labeling Kit 2nd Generation (Cat.#03353575910, Sigma Aldrich, Darmstadt, Germany).

### 2.3. Equipment

Fraction-seq relies on ultracentrifuges to separate the various subcellular fractions from the cytoplasmic fraction. The purity of each subcellular fraction is determined with an automated capillary-based western blot system (ProteinSimple JESS). RNA integrity prior to library preparation is gauged with an Agilent 2100 Bioanalyzer (Agilent Technologies, Santa Clara, CA, USA). Deep sequencing is carried out on the Illumina NextSeq 500, NovaSeq 6000 (Illumina, San Diego, CA, USA), or other equivalent system.

## 3. Fraction-Seq: Fractionation and Deep Sequencing

Fraction-seq combines experimental and analytical techniques to determine the subcellular localization of sncRNAs. The experimental work outlined below provides a detailed, step-by-step workflow for isolating the subcellular fractions of interest, validating their purity, preparing libraries, and performing deep sequencing.

### 3.1. Cell Growth

To obtain total cell RNA, and the cytoplasmic, nuclear, MT, and mitoplast fractions, it is important to collect a number of cells sufficient to execute all subsequent steps. Cells should be grown under standard conditions, and the media should be changed every 2–3 days, depending on the growth rate. The number of dishes to use may vary from 14 to 16, depending on the cell’s morphology. All the dishes should be plated simultaneously to avoid introducing biases.

- Grow cells in 10 cm dishes in complete DMEM media (~16 dishes).
- After cells reach ~100% confluence, wash the plate twice with 2 mL ice-cold 1X PBS and use a cell scraper to scrape the cells.
- Pool the cells in 50 mL ice-cold conical tubes and store them at −80 °C for future use or proceed to the **“Preparation for fractionation”** step.

### 3.2. Preparation for Fractionation

Proper handling of the cells is imperative to maintain the quality of the fractions. The addition of *NaCl* osmotically equilibrates the cells, resulting in slight swelling prior to the addition of the lysis buffer without premature rupture of the MT or the nuclei. Protease inhibitors preserve the endogenous protein complexes.

- Thaw the packed cell volume (PCV) from −80 °C on ice.
- Afterwards, add 1 mL of 0.9% NaCl for every 100 μL of packed cell volume (1:10 volume in mL).
- Vortex the samples for two to three seconds to mix.
- Centrifuge the sample at 500× *g* for 10 min at 4 °C and discard the supernatant.
- Add lysis buffer (+ protease inhibitor + 100 mM EDTA) in 1:1 volume of PCV and resuspend the pellet. The lysis buffer is obtained from *Qiagen Mitochondrial isolation kit (Qiagen #37612)*.
- Transfer the sample to a new 5 mL tube, and put it in a rotor for 10 min at 4 °C.
- Aliquot ~10% of the material remaining after step (vi) to form the “total cell” sample. This will be used to validate fraction purity using the JESS approach (see below).

### 3.3. Cell Fractionation

The subsequent fractionation steps must be carried out in sequential order to preserve the integrity of the fractions and minimize cross-contamination. Once individual fractions are obtained, a small volume of 25 μL should be aliquoted in a microcentrifuge tube for protein validation, while the rest should be stored at −80 °C.

#### 3.3.1. Cytoplasmic Fractionation

- Centrifuge the cell lysate at 1000× *g* for 10 min at 4 °C.
- Remove the supernatant and transfer it to a new 1.5 mL centrifuge tube. This is the ***crude* cytoplasmic fraction.**
- Place the 5 mL containing the pellet tube on ice. This is the ***crude* MT + nuclear fraction**.
- Centrifuge the crude cytoplasmic fraction supernatant at 16,000× *g* for 10 min at 4 °C to remove additional cell debris.
- Transfer the supernatant to a 3 mL ultracentrifuge tube and process it in an ultracentrifuge machine at 100,000× *g* for 30 min at 4 °C.
- Transfer the supernatant to a clean 1.5 mL tube and put it on ice. This is the ***pure* cytoplasmic fraction**.

#### 3.3.2. MT Fractionation

- Using the *Qiagen Mitochondrial isolation kit (Qiagen #37612)*, resuspend the pellet from the above **Cytoplasmic fractionation step (iii**) in a 30× pellet volume of ice-cold disruption buffer (+ protease inhibitor at 100× dilution).
- Vortex the sample briefly to mix.
- Use a 26 G needle and a 1 mL syringe to pass the lysate through the syringe 20 times on ice. Make sure to avoid bubble formation.
- Centrifuge the lysate at 1000× *g* for 10 min at 4 °C.
- Transfer the supernatant to a clean 1.5 mL centrifuge tube and put the pellet on ice for future use (*for nuclear fractionation*).
- Centrifuge the supernatant at 6000× *g* for 10 min at 4 °C, then discard the supernatant. The pellet is the ***crude* MT fraction**.
- In a separate 2 mL round-bottom tube, add 750 μL of ice-cold mitochondrial purification buffer. Carefully add 500 μL of disruption buffer underneath the mitochondrial purification buffer (as shown in the provider’s handbook).
- In the tube containing the pellet (future MT fraction) from above, add 750 μL mitochondrial purification buffer (+7.5 μL PI) and resuspend the pellet by using a pipette.
- Carefully transfer the solution from above to the top of a 2 mL tube containing mitochondrial purification buffer and disruption buffer (as shown in the provider’s handbook).
- Centrifuge the sample at 14,000× *g* for 15 min at 4 °C.
- Remove 750 μL of the supernatant twice so as not to disrupt the pellet. Then resuspend the pellet in the leftover supernatant (~500 μL) and transfer it to a new 1.5 mL tube.
- Add 1 mL mitochondrial storage buffer into the solution and centrifuge at 8000× *g* for 10 min at 4 °C.
- Discard the supernatant and resuspend the pellet in 1.5 mL mitochondrial storage buffer and centrifuge again at 8000× *g* for 10 min at 4 °C. Make sure to measure the volume of the pellet before adding the buffer.
- Repeat step xiii until a clear pellet is visible. At that point, resuspend the pellet in 100 μL mitochondrial storage buffer. This is the ***pure* MT fraction**.

#### 3.3.3. Nuclear Fractionation

- Return to the pellet that was separated for the nuclear fractionation during step (v) of the MT fractionation.
- Measure the volume of this pellet and resuspend it in an appropriate volume of CER I buffer (+ PI) from the NE-PER Nuclear Cytoplasmic Extraction Kit (Thermo Fisher Scientific #78833) (Table 1).
- Vortex the solution at the highest setting for 15 s and incubate the solution on ice for 10 min.
- Add the appropriate volume of ice-cold CER II Buffer (Table 1).
- Vortex the solution at the highest setting for five seconds and incubate it on ice for one minute.
- Vortex the solution at the highest setting again for another 5 s and centrifuge it at maximum speed (~16,000× *g*) for five minutes at 4 °C.
- Discard the supernatant.
- Resuspend the pellet in an appropriate volume of ice-cold NE-PER Buffer (+ PI) and vortex the pellet at the highest setting for 15 s followed by incubation on ice for 10 min.
- Centrifuge the tube again at the maximum setting (~16,000× *g*) for 10 min at 4 °C.
- Transfer the supernatant to a clean pre-chilled tube. This is the ***pure* nuclear fraction**.

#### 3.3.4. Mitoplast Fractionation

- Divide the MT fraction obtained above into thirds.
- Transfer 2/3rds into new tubes. This is the ***pre-mitoplast fraction***.
- Centrifuge the pre-mitoplast fraction at 8000× *g* for 10 min and discard the supernatant.
- Resuspend the pellet in 10× pellet volume 100 mM swelling buffer (NaPO_4_, pH 7.4 + PI) and incubate on ice for 20 min.
- Centrifuge the solution at 12,000× *g* for 15 min and discard the supernatant.
- Wash the pellet in 1 mL mitochondria storage buffer (+ PI).
- Resuspend the ***pure* mitoplast fraction** in 50 μL mitochondrial storage buffer (+PI).

### 3.4. Measuring Protein Concentration

Prior to RNA extraction, the purity of fractions from different cellular compartments must be evaluated with a protein assay to gauge cross-fraction contamination. As stated, the absence of contamination at this point would be limited by the sensitivity of this assay. To this end, protein concentration is quantified using the *Bradford protein* assay and Simple Western^TM^ technology, a capillary-based western immunoassay performed using a JESS automated western blot system (ProteinSimple, *Bio-Techne #004-650*). The cellular fractions (cytoplasmic, MT, and nuclear) obtained above are subjected to *BioRad Bradford Protein Assay* (Cat.# 5000006, Bio Rad, Hercules, CA, USA) to calculate the concentration of protein.

- Mix the Bradford reagent with water at a 1:5 ratio.
- Pipette 200 μL of diluted Bradford reagent into a 96-well plate.
- Pipette 4 μL of each fraction sample along with their technical replicate to each Bradford reagent and mix them.
- The blank consists of 4 μL each of lysis buffer, mitochondrial storage buffer, and NE-PER buffer. Pipette these into the wells of the 96-well plate.
- Incubate the plate at room temperature for 10 min.
- Measure the protein concentration.

#### Simple Western (JESS)

Use a Simple Western size-based assay to form ProteinSimple (JESS) to validate the purity of cell fractions based on protein profiling.

- Take the tubes out of the EZ Standard packet (pink, green, and white). The pink tube contains 5X fluorescent master mix, the white tube contains DDT, and the green tube contains biotinylated ladder.
- Add 40 μL of deionized water to the white tube to make a 400 mM solution, and mix.
- Add 20 μL of 10× sample buffer and 20 μL of prepared 40 mM DDT from above to the pink tube, and mix.
- Add 20 μL of deionized water to the green tube and mix.
- In a new tube, combine 200 μL luminol-S and 200 μL peroxide, and put it on ice.
- Dilute the fraction samples to 0.2 μg/μL in 0.1× sample buffer.
- Pipette 5× the fluorescent master mix made above with the diluted fraction samples in a 5:1 ratio.
- Vortex the mixture briefly and denature the sample at 95 °C for five minutes in a PCR machine.
- Following denaturation, vortex the samples and briefly centrifuge them.
- Place the samples on ice until further use.
- Dilute primary antibodies in Antibody Diluent 2 Buffer according to the dilution shown in Table 2.
- The secondary antibodies that we will use are anti-rabbit (*ProteinSimple #DM-001*) and anti-mouse (*ProteinSimple #DM-002*) antibodies. They do not need to be diluted.
- Pipette the samples in 12–230 kDA JESS/WESS Separation Module, 8 × 25 Capillary Cartridge (*ProteinSimple #SM-W004*) according to the figure provided in the manufacturer’s protocol. Use sample buffer to block the empty lane.
- After the addition of all required items, centrifuge the plate at 5000× *g* for five minutes at room temperature.
- Set up the WES machine and start the run.

### 3.5. RNA Extraction

High-quality RNA is extracted from each subcellular fraction in order to profile the subcellular localization of small RNAs using RNA-seq and to validate the distribution of the small RNAs using northern blotting.

- Transfer 500 μL of the fraction samples to a 1.8 mL Eppendorf tube.
- Add 1 mL TRIzol (*TRIzol^®^ Reagent #15596-018*) and 200 μL chloroform into the tube containing the samples and mix them by pipetting up and down.
- Centrifuge the samples at 16,000× *g* for 15 min at 4 °C.
- Transfer the aqueous phase of the sample into a new 1.8 mL tube and add an equal volume of isopropanol into each tube.
- Precipitate the tubes at −20 °C for 20 min.
- After precipitation, centrifuge the tubes at 16,000× *g* for 10 min at 4 °C.
- Discard the supernatant using vacuum suction or a pipette.
- Add 250 μL of 75% ethanol into each tube to wash the pellet and vortex them briefly to mix the pellet.
- Centrifuge the samples at 16,000× *g* for five minutes at 4 °C.
- Discard the supernatant and air-dry the pellet on ice for at least 15 min to remove all the leftover ethanol.
- Resuspend the pellet in 15 μL of molecular-grade water (*Fisher #BP2819-1*) and vortex the tube to mix the pellet.
- Measure the RNA concentration using a spectrophotometer.

### 3.6. Northern Blot

Northern blotting can provide independent evidence of the purity of the subcellular fractions, where certain small RNA species are enriched in one fraction and absent from others. Additionally, it can be used at the end of Fraction-seq to validate experimentally the subcellular localization of select sncRNAs.

#### 3.6.1. Gel Preparation

- For 10 mL gel preparation, aliquot 3.75 mL of 40% acrylamide into 50 mL tube.
- Add 1 mL 10X TBE buffer into the same tube.
- Weigh 4.2 g of urea and pour it into the 50 mL tube.
- Bring up the volume of the tube to 10 mL by adding water to bring down the concentration of 10X TBE to 1X TBE.
- Put a small magnet inside the tube and stir it upside down on a stir plate to mix the contents of the tube.
- While the urea is getting mixed, set up a gel cast.
- After the urea is completely dissolved, add 150 μL of 10% ammonium persulfate and 10 μL of TEMED and vortex the tube.
- Pour the gel into the 1.5 mm molds using a pipette and let it sit for 15 to 20 min.

#### 3.6.2. Calculating Cell Fraction Percentage for Northern Blot

- For the total fraction, divide the total volume taken at the beginning by the starting volume post addition of the lysis buffer (~10%).
- For the cytoplasmic fraction, divide the final supernatant volume by the initial volume after the addition of the lysis buffer and multiply by ~90% (~67.5%).
- For the MT and nuclear fractions, we account for ~10% loss of material. The pure MT fraction is equivalent to all the MT fractions post removal of other organelles (~80%).
- Scale the cell fractions by the total cell volume to obtain equivalents.

#### 3.6.3. Sample Preparation

- Calculate the cell fraction equivalents and standardize the samples to 1 μg total RNA.
- Vortex the samples and put them on a heat plate set at 70 °C for five minutes.
- Submerge the gel in 1X TBE buffer.
- Load 5 μL of DIG dye, followed by 5 μL of sample.
- Run the gel at 250 V for 45–50 min (until the dye has reached the bottom of the gel).

#### 3.6.4. Probe Preparation

- Thaw buffers 1, 2, 3, and 4 from the DIG oligonucleotide labeling kit (*DIG 3′-End Labeling Kit, 2nd Gen: 3353575910*).
- Label four 1.5 mL centrifuge tubes according to the probe to be added. U1, U2, LysCTT tRNA, and MT ValTAC tRNA are the probes to be diluted (Table 3). U1 and U2 serve as nuclear markers, whereas LysCTT tRNA and ValTAC tRNA serve as markers for the cytoplasmic fraction and MT fraction, respectively.
- Dilute each probe to 10 nM (original concentration is 100 μM) by adding 9 μL MilliQ water to 1 μL probe.
- Add 4 μL of buffers 1 and 2 and 1 μL of buffers 3 and 4 into each tube containing diluted probes.
- Vortex the tubes briefly.
- Place the tubes in a water bath set at 37 °C for 15 min.
- After the incubation period, add 2 μL of 0.2 M, pH 8 EDTA into each tube. This will bring the volume up to 22 μL.
- Put the prepared probes on ice until further use.

#### 3.6.5. Membrane Transfer

- After the completion of the gel run, in a plastic case, pour some volume of 1X TBE from the cast in which the gel was running.
- Get a Hybond™- N^+^ membrane (*Amersham Bioscience #RPN303B*) and soak it in 1X TBE buffer along with two blotting papers inside the cast prepared above.
- Sandwich the gel between the two blotting papers, with the membrane placed underneath the gel, and run the transfer at 0.4 A for 20–25 min.
- After the transfer (the gel should not have any visible dye on it), remove the membrane and label the membrane appropriately using a pencil.
- Air-dry the membrane for at least five minutes.
- Cross-link the membrane at 120,000 μJ/cm^2^.
- After completing the cross-link, obtain a cylindrical flask and pour 10 mL of hybridization buffer (*PerfectHyb™ Plus Hybridization Buffer: H7033-1L*) into it.
- Put the membrane inside the flask and pre-hybridize the membrane for 30 min, rotating at 37 °C.
- In the meantime, obtain three more conical flasks and label them according to the probes to be used.
- After 30 min, take the flask out of the rotor machine and discard the hybridization buffer.
- Cut the membrane using a fresh blade according to where you expect to find your targets for the probes and place them inside four different conical flasks.
- Aliquot 5 mL of hybridization buffer into each conical flask and load 2 μL of the respective probes prepared above.
- Place the flasks inside the rotor machine for overnight incubation.
- Thaw blocking buffer solution at 4 °C.

#### 3.6.6. Imaging

- Prepare wash buffer 1 by mixing 4 mL of 20X SSC, 36 mL MilliQ water, and 400 μL of 10% SDS in a 50 mL tube. The final concentration of SSC and SDS should be 2× and 0.1% respectively.
- Discard the hybridization buffer from the previous day, pour 10 mL of wash buffer into each flask, and incubate them in the rotor machine for five minutes.
- Repeat steps (i) and (ii) again and turn up the speed of the machine to 3.
- Prepare wash buffer 2 by mixing 1 mL SSC, 39 mL MilliQ water, and 400 μL of 10% SDS (0.5× SSC and 0.1% SDS).
- After five minutes, discard the old wash buffer, pour 10 mL of the new wash buffer, and spin again for 15 min.
- Repeat steps (iv) and (v).
- In the meantime, prepare wash buffer 3 by mixing 4 mL of DIG Wash and Block Buffer Set #1 (BUFFER 1) with 36 mL MilliQ water.
- After 15 min, remove the membranes from their respective flasks and place them on a clear plastic tray with the labeled side facing upward. Add 10 mL of wash buffer 3 and shake for five minutes on a shaker.
- In the meantime, make a blocking buffer by adding 4 mL of DIG Wash and Block Malic Acid Buffer #2 (BUFFER 2) and 4 μL of blocking buffer solution (thawed the day before). Bring the volume to 40 mL with MilliQ water.
- After five minutes, discard wash buffer 3 and pour 10 mL of the blocking buffer prepared in step (ix), and rock the plates for 30 min.
- Prepare a new batch of blocking buffer by repeating step (ix) and add 4 μL of DIG antibody into this newly prepared blocking buffer (1 μL antibody per 10 mL blocking buffer).
- After 30 min, discard the blocking buffer from before, pour 10 mL of the newly prepared blocking buffer (+ DIG antibody), and rock the plates for 30 min.
- In the meantime, prepare wash buffer 3 again by repeating step (vii).
- After 30 min, discard the blocking buffer (+ DIG antibody) and pour 10 mL of wash buffer 3 into each plate.
- Wash the membrane with wash buffer 3 for 15 min. Discard wash buffer 3, then repeat the washing step using wash buffer 3 once more.
- In the meantime, prepare the detection buffer by mixing 4 mL 10× detection buffer 4 from the DIG kit with 36 mL MilliQ water (1× detection buffer).
- After 15 min, discard wash buffer 3 and incubate the membranes in 10 mL detection buffer 4 for six minutes.
- Discard the detection buffer. Remove any excess buffer with a paper towel.
- Align the membrane on a piece of cellophane and pipette 600 μL of oxidizing reagent on the membrane (make sure the entire membrane is covered by the reagent). Incubate for five minutes.
- Take images of the membrane at high sensitivity/resolution. Confirm the purity of the fractions (to the extent afforded by the sensitivity of northern blotting).

### 3.7. Library Preparation

Extracted RNA from each subcellular fraction must be converted into sequencing-compatible libraries to allow for high-throughput, quantitative analysis of the corresponding sncRNAs. We recommend that library preparation be performed using the NEBNext Small RNA Library Prep Set for Illumina (*#E7330*) according to the manufacturer’s protocol.

- Assess RNA integrity and purity using the Agilent 2100 Bioanalyzer. Use 100 ng of RNA per subcellular fraction as input.
- Perform 3′ adapter ligation followed by 5′ adapter ligation.
- Perform reverse transcription using adapter-specific primers, followed by PCR amplification.
- Perform library quality control and size selection of sncRNAs to enrich for sncRNAs (payload) in the 18–50 nt range using 6% polyacrylamide gel. The library obtained will be used for sequencing.

### 3.8. Deep Sequencing

Deep sequencing enables high-throughput, quantitative measurement of sncRNAs across different subcellular compartments while permitting differentiation among sncRNAs with very similar sequences. It can be performed on an appropriate platform, such as the Illumina NextSeq 500 or NovaSeq 6000, following the respective manufacturer’s protocol. All samples should be sequenced at a minimum average depth of 30 million reads, using single-end, 75-base reads.

## 4. Fraction-Seq: Mixed-Effects Modeling and Analysis

At the end of the deep sequencing portion of the protocol, we will have generated detailed profiles of sncRNAs across multiple biological replicates of the nuclear, cytoplasmic, whole MT, and mitoplast fractions of the cell line being studied, as well as of total RNA. It is important to note two things here. First, each fraction will have been profiled at different depths through sequencing. This results from the fact that even though the inherent molecular diversity differs among fractions, they are all profiled at a similar depth, by design. Consequently, two equi-abundant sncRNAs, each from a different fraction, would not be equi-abundant when examined in total RNA. Second, the fractionation step is bound to introduce cross-fraction contamination. This means that the measured abundance of some sncRNA(s) in a fraction of interest will be inflated because of contamination by another fraction in which the sncRNA(s) is/are naturally present. While the contribution of cross-contamination to the measured abundance may be low, it can be consequential and must be accounted for and removed during the analysis. This is precisely what the mixed-effects modeling achieves.

### 4.1. Mixed-Effects Modeling

The abundance of each sncRNA in a fraction that the sequencing step determines is the sum of its true abundance in that fraction, contributions from other contaminating fractions, and contributions from replicate-specific technical errors. This step models the underlying potential cross-fraction contamination technical errors and uses the resulting estimates to recover the true abundance of each sncRNA in each fraction.

#### Specifics of the Model

In the original study, we focused on three categories of sncRNAs: miRNAs/isomiRs, tRFs, and rRFs [3]. This made it easier to draw comparisons to previous reports in the area of breast cancer, which was the theme of the study. However, the mathematical model is sncRNA-class agnostic, making it applicable to any type of sncRNA. The R code implementation of the method makes this more evident. Users need only assign a unique label to each distinct sncRNA sequence; to facilitate this step, we also provide an automated solution, which we describe in The “License Plates” Approach section below.

Many of the sncRNAs discussed here are co-expressed “siblings” that arise from the same parental RNA, e.g., isomiRs from the same arm of a miRNA, or tRFs from the same isodecoder. The model treats siblings as independent from one another. Moreover, the model accounts separately for the contributions by each contaminating fraction and each biological replicate to each sncRNA’s measured abundance. The model considers the cytoplasmic fraction to be “sufficiently pure.” On the other hand, the nuclear, MT, and mitoplast fractions are generally assumed to be contaminating each other. Lastly, the model assumes that an sncRNA’s measured abundance in total RNA is approximately equal to the weighted sum of its reconstructed abundances in the nuclear, cytoplasmic, and MT fractions, with the weights being determined by the model. By weighting each fraction separately, we account for the varying degrees of material loss during fractionation. The formulas remain as in the original publication [3], apply to all types of sncRNAs, and are not reproduced here. In other words, the “*i*-th molecule” can belong to any sncRNA class.

### 4.2. Processing the FASTQ Files

Each biological replicate will generate five FASTQ files, one each for the nuclear, cytoplasmic, MT, and mitoplast fractions and total RNA. The sequenced reads in each file are processed as follows:

- The reads are quality trimmed, then have their 3′-adapters removed. For this step, we recommend using the *cutadapt* utility [49].
- Any processed reads whose length is not at least 18 nts are excluded from further processing as their genomic origin cannot always be determined unambiguously [2,50,51].
- Independent of the category to which sncRNAs belong, only those with a reads-per-million (RPM) abundance ≥ 10 in each fraction are kept for further processing. RPM is calculated by dividing the copy number of unique sequences by the total number of sequenced reads and multiplying by one million.
- We identify and exclude from further processing all sncRNAs whose abundance exceeds threshold in the mitoplast fraction but not the corresponding MT fraction. We apply this computational filter because we do not treat the mitoplast fraction with RNase A due to its limiting yield.

### 4.3. Optionally Assigning sncRNAs to the Various sncRNA Categories

Before embarking on the analysis, all sncRNAs whose average exceeds threshold in at least one fraction and in total RNA must be given unique identifiers. We present two possibilities: (a) using “license plates” and (b) using category-specific tools/labels.

#### The “License Plates” Approach

We strongly recommend this approach because it is easy to use and applies to all sncRNA categories. Here, each sncRNA is assigned a license plate, a unique, sequence-dependent, genome-assembly-independent identifier. License plates represent a one-to-one mapping from a nucleotide sequence to a label, and, therefore, are invertible. We first introduced license plates to label tRFs [52] and have since extended them to isomiRs [2], rRFs [6], yRFs [8], and also unclassified molecules [9]. License plates obviate the need for brokers who communicate labels at irregular intervals through releases of public databases, thereby solving a critical problem in this field. This approach enables anyone to label their favorite sncRNA instantly, while ensuring that scientists from different laboratories and countries assign the same label to the same sequence. We make codes for ‘minting’ license plates freely available through the GitHub page of Jefferson’s Computational Medicine Center at https://github.com/TJU-CMC-Org/MINTplates.

As an alternative to license plates, one can use any tool that can process FASTQ files and recognize in them miRNAs/isomiRs, tRFs, rRFs, and yRFs. It is critical that the used tool be able to distinguish among isomiRs, non-templated isomiRs, and exclusive or ambiguous tRFs to prevent double-counting. We recommend using the tools and methodologies we developed previously, which satisfy these requirements. For miRNAs/isomiRs, we recommend using isoMiRmap [2], which can distinguish among canonical miRNAs, non-canonical isomiRs, and non-templated isomiRs. For tRFs, we recommend using MINTmap [50], which can distinguish between nuclear-genome- and MT-genome-derived tRFs and identify those that are ambiguous in origin. For rRFs and yRFs, we recommend using the methods we published previously [6,8,10].

### 4.4. Analysis and Open-Source Codes

At this point, the FASTQ files for all fractions and total RNA from all biological replicates have been pre-processed, and unique identifiers assigned to each sncRNA with a minimum recommended length of 18 nts and a minimum recommended abundance of 10 RPM.

The final step before embarking on the analysis is to create a table with tab-separated entries. The number of rows should be equal to the union of all sncRNAs across total RNA and the four fractions that satisfy the length and abundance constraints. The number of columns should be equal to 1 + 5*N, where N is the number of biological replicates. The first column lists the unique identifiers for all sncRNAs. Here, we recommend using license plates. Each group of five columns that follows should correspond to measured RPM values in the *k*-th replicate (k ≥ 3) for total RNA and the cytoplasmic, nuclear, MT, and mitoplast fractions, in that order; i.e., columns 2–6 correspond to biological replicate 1, columns 7–11 correspond to replicate 2, and so on. The (i,j) cell of this matrix lists the normalized abundance (in RPM) of the *i*-th sncRNA in the fraction or total RNA for the biological replicate that corresponds to the *j*-th column.

What remains is to use the codes we provide to process the tab-separated table. Upon completion, the codes will generate the recovered true abundances for each sncRNA in each fraction under the constraint that the sum of the abundances across the nuclear, cytoplasmic, and MT fractions of the same biological replicate approximates the value of 1 million, and also the sncRNA’s abundance in total.

As stated above, we ported the original SAS codes to R to facilitate Fraction-seq’s use by other groups. The codes are freely available on the website Jefferson’s Computational Medicine Center, maintained on GitHub at https://github.com/TJU-CMC-Org/Fraction-seq.

## 5. Fraction-Seq: Discussion and Conclusions

It was previously thought that parental RNAs, including miRNA precursors, tRNAs, and rRNAs, give rise to the *same* sncRNA products in all settings. Moreover, it was believed that the genome—nuclear or MT—encoding these parental RNAs determines the sncRNAs’ subcellular localization and that the latter is fixed across tissues and diseases.

Recent studies by others and us have shown multiple exceptions to these assumptions. We now know that a parental RNA simultaneously produces multiple sibling sncRNAs [53] whose abundance and exact sequences depend on tissue type and disease [1,6,25] and personal attributes (e.g., sex, ancestry, age) [21,22,23,27]. We also know that the localization of an sncRNA within the cell depends on its sequence [3,31,32] and on the cell type [3]. Despite these very intriguing dependencies, most sncRNAs remain uncharacterized.

A first corollary of these findings is that cell lines from different donors will express different sncRNAs, even if they model the same disease. A second corollary is that sibling sncRNAs will localize differently within the cell and, thus, have different functions, even though their sequences can be very similar. Consequently, mapping the location within the cell of sncRNAs will greatly benefit studies aimed at functionalizing them. This is because constraining their location also constrains the pool of their possible targets. The recency of these findings means that previous functionalization attempts were conducted without this key information and, thus, may have described events that do not occur endogenously.

Having these maps can help avoid unwarranted experimentation by providing a framework and a resource for designing molecular studies. It will also accelerate the generation of foundational results by characterizing the many members of the recently discovered classes of tRFs, rRFs, and yRFs. Moreover, by restricting the pool of potential targets to only those mRNAs and long non-coding RNAs (lncRNAs) that co-localize with the sncRNA, we anticipate that future functionalization efforts will reveal currently unsuspected linkages between regulatory sncRNAs and their effectors. Given the dependence of isomiRs, tRFs, rRFs, and yRFs on sex, ancestry, cell/tissue type, disease, and survival, the availability of these maps can also guide basic research towards uncovering the molecular events that underlie disparities by sex or ancestry; can facilitate disease stratification; and can help discover better biomarkers for diagnosis and prognosis and better-tuned therapeutics.

Two points complicate the creation of these maps by simply using fractionation followed by deep sequencing. First, multiple sncRNAs from different categories have been shown to be present in unexpected places (reviewed in the Section 1). Second, it is difficult to obtain pure subcellular fractions, primarily because of cross-fraction contamination.

To put our work in context, we note that methods combining biochemical cell fractionation with deep sequencing followed by computational analyses of the RNAs found in the fractions date back many years [43,54,55], with most methods focusing on long non-coding RNA (lncRNAs). These methods typically relied on northern blotting, western blotting, or qRT-PCR methods to ensure high fraction purity. However, the inevitable cross-fraction contamination hampered the quantification of these RNAs. Fraction-seq solves the problem using a rigorous mathematical model that first estimates cross-fraction contamination separately for each RNA and then uses these estimates to adjust the abundance of each RNA in a fraction.

Decidedly, the most comprehensive study of this kind is that of Djebali et al. [43], who studied the RNA contents of the nucleus, cytoplasm, and whole cell in multiple cell lines. RNAs were separated by length into two groups: those with length ≥ 200 nts and the remainder, and the groups were sequenced separately. Fractionation was carried out according to standard protocols, suggesting that some level of cross-fraction contamination likely remained. Because the group of the shorter RNAs included RNAs from several different classes, microRNAs were comparatively underrepresented, which may have contributed to subsequent analyses focusing on lncRNAs instead. Among those, the LncAtlas study is more prominent [56]. LncAtlas introduced the relative concentration index (RCI), which is the base-2 logarithm of the ratio of an lncRNA’s normalized abundance (in fragments per kilobase per million mapped reads) in the cytoplasm over that in the nucleus. This ratio essentially captures enrichment of the lncRNA in the cytoplasm (RCI > 0) or the nucleus (RCI < 0). In subsequent work, the authors provided a comprehensive review of various measures for estimating compartment-specific enrichments and highlighted the problems faced by approaches that rely on relative abundances [57].

More recently, a slightly different method that relied on relative abundances was described by Dai et al. [58]. Their approach uses the transcript abundances in the nucleus, cytoplasm, and whole cell to estimate the relative contributions of the two compartments. The study’s results were generally consistent with previous estimates; however, it, too, only considered lncRNAs and mRNAs and did not reconstruct each molecule’s abundance in the examined compartments.

The earliest related work on sncRNAs of which we are aware [59] examined the miRNA and tRF composition of the nuclear and cytoplasmic fractions from the human nasopharyngeal carcinoma 5-8F cell line. The study reported that most of the miRNAs found in the cytoplasm are also in the nucleus, suggesting translocation. However, because the community had not yet realized the importance of isomiRs, the assessment was made without distinguishing among isomiRs from the same locus. Figure 5 of our original report [3] underscores the importance of making that distinction by showing that canonical, non-canonical, and non-templated isomiRs from the same miRNA arm can localize *differently* in the same cell line. A few design choices of the study by Liao et al. [59] were more consequential. First, the study evaluated the purity of fractions using northern blots, which have limited sensitivity. Second, the study’s cytoplasmic fraction included the mitochondrial organelle, as evidenced by the northern blot in Figure 1B of their study, which shows the MT tRNA^Val^ in the cytoplasmic fraction. Additionally, the study did not explicitly consider how cross-fraction contamination could influence their findings, nor did it attempt to reconstruct the abundances of the various sncRNAs in the fractions, which is one of the contributions of Fraction-seq.

A more recent study [60] also examined sncRNA localization in the nucleus and the cytoplasm. This study focused exclusively on miRNAs and did not consider isomiRs. A novel element of the study was the addition of a spike-in oligonucleotide in the nuclear and cytoplasmic fractions that allowed abundance normalization prior to the computational analyses. This study also computed and reported differential abundances between the two compartments and did not attempt to reconstruct the actual abundances by estimating cross-fraction contamination. Interestingly, the study found that miRNAs from the 3p arm of let-7 family members are almost exclusively present in the cytoplasm, which agrees with our findings [3] in the BT-20 cell line. However, as we have shown, in MDA-MB-231 cells, isomiRs from these arms are primarily in the nucleus and the MT, whereas they are not produced in MDA-MB-468 at levels exceeding our 10 RPM threshold. These differences underscore the cell-type dependence of sncRNA localization.

Finally, APEX-seq [61,62] is a different approach. It leverages the knowledge that certain proteins localize to a region of interest, then tags them with a genetically encoded engineered ascorbate peroxidase (APEX2) that promotes biotinylation of proteins and RNAs in its immediate vicinity (the latter measured in nanometers). The biotin-tagged RNAs can then be isolated and sequenced to generate a profile of the RNAs that are present in the region of interest. Two factors that complicate the use of this method include the need to engineer the protein(s) of interest and its applicability to living cells. While there are no intrinsic constraints that prevent the method from profiling isomiRs, tRFs, rRFs, and yRFs in the regions of interest, we are not aware of such reports.

The Fraction-seq [3] method we presented differs from these earlier efforts in recognizing that cross-fraction contamination is unavoidable. With that in mind, we designed an integrated experimental and analytical framework for creating accurate sncRNA localization maps by identifying the contributions of contamination and technical errors to an sncRNA’s abundance and removing them to recover its true abundance. Fraction-seq determines and applies such corrections separately for each sncRNA and fraction. As evidenced from the description of Fraction-seq, the method is independent of and can be applied to any adherent cells from any organism, and can reconstruct the abundance of all categories of sncRNAs. Fraction-seq can potentially be applied to bulk tissues as well, but this remains to be validated.

## 6. Fraction-Seq: Limitations

The experimental portion of Fraction-seq relies on the quality of the kits obtained from the respective companies. Based on our experience, the quality of the kit may occasionally influence the quality and quantity of the fractions, particularly the MT and mitoplast ones. It is therefore recommended that the same batch of reagents be used to process all biological replicates from a given source. While automated ProteinSimple JESS can be used to quantitatively and quickly assess fraction purity, the system may not be readily available; in this case, standard western blotting is an adequate alternative.

## Figures and Tables

**Table 1:** Reagent volumes for different packed cell volumes * (reproduced from the Thermo Fisher NE-PER^TM^ Nuclear and Cytoplasmic Extraction Reagents kit protocol).

Packed Cell Volume (μL)	CER I (μL)	CER II (μL)	NER (μL)
10	100	5.5	50
20	200	11	100
50	500	27.5	250
100	1000	55	500

* 2 × 10^6^ cells are roughly equivalent to 20 μL packed cell volume.

**Table 2:** Antibodies used for the western blotting step.

Name	Target	Dilution	Ab Target
GAPDH (NEB #2118)	Cytoplasm	1:600	Rabbit
SDHA (NEB #11998)	MT and Mitoplast	1:200	Rabbit
TFAM (NEB #8076)	MT and Mitoplast	5:45	Rabbit
Lamin A/C (NEB #4777)	Nucleus	1:400	Mouse
Cytochrome C (BD Pharmagen #556433)	Mitoplast	1:50	Mouse

**Table 3:** Northern blot probe sequences.

Nuclear RNA Marker U1	GTGATCATGGTATCTCCCCTGCCAG
Nuclear RNA marker U2	CCAACTCCTAGTTCCAAAAATCC
Cyto RNA marker tRNA LysCTT	GTCTCATGCTCTACCGACT
Mito RNA marker tRNA ValTAC	GTGTTAAGCTACACTCTG

## Data Availability

The codes are freely available through our center’s GitHub page at https://github.com/TJU-CMC-Org/Fraction-seq.
